# Comparison of efficacy and safety of second‐ and third‐generation TKIs for non‐small‐cell lung cancer with uncommon EGFR mutations

**DOI:** 10.1002/cam4.6229

**Published:** 2023-06-12

**Authors:** Yue Hao, Manyi Xu, Jianan Jin, Jinfei Si, Chunwei Xu, Zhengbo Song

**Affiliations:** ^1^ The Second Clinical Medical College of Zhejiang Chinese Medical University Hangzhou China; ^2^ Department of Clinical Trial, Zhejiang Cancer Hospital Hangzhou China; ^3^ Department of Respiratory Medicine, Jinling Hospital Nanjing University School of Medicine Nanjing China

**Keywords:** afatinib, efficacy, NSCLC, osimertinib, safety, uncommon EGFR mutation

## Abstract

**Background:**

The efficacy of definite for non‐small‐cell lung cancer (NSCLC) with uncommon epidermal growth factor receptor (EGFR) mutations has been preliminarily demonstrated. However, there is a paucity of data with which to compare the efficacy and safety of second‐ and third‐generation TKIs in patients with NSCLC carrying uncommon EGFR mutations.

**Methods:**

We compared the efficacy and safety of second‐ and third‐generation TKIs in all NSCLC patients in whom next‐generation sequencing confirmed uncommon EGFR mutations, including G719X, S768I, and L861Q. The parameters analyzed included the objective response rate (ORR), disease control rate (DCR), progression‐free survival (PFS), and overall survival (OS). The rate of treatment‐related adverse events (AEs) reflected the safety of these TKIs.

**Results:**

Eighty‐four NSCLC patients with uncommon EGFR mutations were enrolled between April 2016 and May 2022 at Zhejiang Cancer Hospital, including 63 treated with second‐generation TKIs and 21 treated with third‐generation TKIs. The ORR for all patients receiving TKIs was 47.6%, and the DCR was 86.9%. The median PFS for NSCLC patients with uncommon EGFR mutations receiving TKIs was 11.9 months and OS was 30.6 months. There was no significant difference in PFS after treatment with second‐ or third‐generation TKIs (13.3 vs. 11.0 months, respectively, *P* = 0.910) or in OS (30.6 vs. 24.6 months, respectively *P* = 0.623). The third‐generation TKIs showed no severe toxicity.

**Conclusions:**

The efficacy of second‐ and third‐generation TKIs for NSCLC with uncommon EGFR mutations does not differ, and so can be used to treat NSCLC patients with these mutations.

## 1 INTRODUCTION

Epidermal growth factor receptor (EGFR) is one of the commonest mutation sites in patients non‐small‐cell lung cancer (NSCLC), and accounts for 30%–60% of all mutations in these patients in Asia.^1^ Tyrosine kinase inhibitors (TKIs) of EGFR are regarded as the standard therapy regimen for NSCLC patients, and the first‐ and second‐generation TKIs have shown efficacy beyond that of platinum‐based chemotherapies for cancers with various mutations, including some with rare mutations.^2^, ^3^, ^4^, ^5^, ^6^ For these rare types of mutations, the results of the AURA3 clinical trial indicated that osimertinib showed greater efficacy and lower toxicity than platinum–pemetrexed therapy after a first‐line EGFR‐TKI therapy for NSCLC with the EGFR T790M mutation.^7^ The efficacy of osimertinib was also demonstrated in NSCLC patients with uncommon EGFR mutations.^8^

With the development of next‐generation sequencing (NGS), the technology available to detect cancer mutations has become more convenient and more precise. The mutations in cancer‐associated genes can be divided into two groups: common and uncommon.^9^ In both groups, the sensitive mutation was contained with two types. Previous studies have reported several sensitive mutations among the uncommon mutations in EGFR, including G719X, S768I, and L861Q, which have preliminarily shown responsiveness to both afatinib and osimertinib, with better brain infiltration in NSCLC patients.^6^, ^8^ Compared with the second‐generation TKIs, the efficacy of the first‐generation TKIs not manifested the surprising results.^10^, ^11^, ^12^, ^13^ Later, afatinib had been approved by U.S. Food and Drug Administration as the standard therapy regimen for NSCLC patients with metastasis and uncommon mutations, including G719X, S768I, and L861Q, for which the efficacy and benefit of second‐ and third‐TKIs have been demonstrated.

However, there is a paucity of data with which to compare the efficacy and safety of second‐ and third‐generation TKIs for NSCLC with uncommon EGFR mutations. Therefore, in this study, we compared the efficacy and safety of second‐ and third‐generation TKIs for NSCLC with uncommon EGFR mutations, which may provide new therapeutic options and strategies in clinical practice.

## 2 METHODS

### 2.1 Patient eligibility

The study group included NSCLC patients diagnosed at Zhejiang Cancer Hospital between April 2016 and May 2022. All patients presented with Stage III (with no opportunity to receive surgery or radiotherapy) or Stage IV disease, which was confirmed with computed tomography (CT), bone scanning, or magnetic resonance imaging (MRI). Both untreated patients without receiving systematic treatment and those suffered the failure of chemotherapy could be included in current study. The performance status of the patients, who had Eastern Cooperative Oncology Group performance status scores (PS) ≤2, indicated that they could tolerate adverse events (AEs). The liver and kidney function sustained the better condition, and did not influence the therapy of the patients. The information recorded for all patients included the diagnostic process, treatment, response, and disease progression. Rare EGFR mutations involving single mutations or complex mutations, such as G719X, S768I, and L861Q, were included in the analysis. All gene mutations were detected with NGS. The DNA is detected from the tissue biopsy or blood samples. The detection of patients was involved on the Next sequence 500 (Illumina, San Diego, CA) with paired‐end reads with target sequencing depth of 1000× and 10,000× for tissue and plasma samples, respectively, which is using commercially available panel targeting genes. The exclusion criteria for the study were abnormal bone marrow function, severe basic disease, and active infection requiring systemic therapy. The protocol was approved by the Institutional Ethics Committee at Zhejiang Cancer Hospital. The study was performed in accordance with guidelines of the Helsinki Declaration (revised in 2013), and individual patient consent for the study was waived.

### 2.2 Treatment methods

All the NSCLC patients received second‐ or third‐generation TKIs according to the guidelines. The dose of afatinib was 40 mg a day, but with the occurrence of diarrhea or rash and pruritus, the dose of afatinib was reduced to 30 mg a day. The dose of osimertinib was 80 mg a day. Other types of the third‐generation TKIs, such as almonertinib or furmonertinib, were also included in the analysis, at doses of 110 mg or 80 mg a day, respectively. If severe AEs occurred in the patients, treatment was stopped.

### 2.3 Responses and toxicity

Physical examinations and laboratory tests were performed every 3 weeks. The treatment response was assessed every 6 weeks (every two cycles of treatment), according to the criteria of RECIST 1.1. The responses to treatment included a complete response (CR), partial response (PR), and stable disease (SD). Progressive disease (PD) was the only classification of nonresponders. The objective response rate (ORR) was defined as CR + PR. The disease control rate (DCR) was defined as CR + PR + SD. The evaluation of safety and toxicity were evaluated with the National Cancer Institute Common Terminology Criteria for Adverse Events (version 4.03).

### 2.4 Follow‐up

The survival analysis included progression‐free survival (PFS) and overall survival (OS), and the follow‐up timing was defined regularly. PFS encompassed the time from the first dose of second‐ or third‐generation TKI until disease progression or death from any cause. OS was defined from the first dose of second‐ or third‐generation TKI to death from any cause or the last follow‐up. The date of last follow‐up was September 5, 2022.

### 2.5 Statistical analysis

PFS and OS were analyzed with the Kaplan–Meier method and the different groups were compared with a log‐rank analysis. The statistical analysis was two‐sided, and *P* < 0.05 was considered statistically significant. The statistical package for the social sciences (SPSS) for Windows version 25 (SPSS, Chicago, IL) and GraphPad Prism (version 9) were used for all statistical analyses.

## 3 RESULTS

### 3.1 Patient characteristics

In total, 84 patients received second‐ or third‐generation TKIs at Zhejiang Cancer Hospital between April 2016 and May 2022. The characteristics of all patients with uncommon EGFR mutations are included in Table 1. All the NSCLC patients who received TKI therapy had a good performance status, with PS scores ranging from 0 to 1. In this study, the median age of the patients was 62 years (range 29–81) and 53.6% (*n* = 45) were male (female, *n* = 39). The majority patients remained in stage IV (*n* = 80). Thirty‐nine patients had a history of smoking and 34 (40.5%) suffered brain metastasis. Sixty‐three patients received second‐generation TKI therapy and 21 received the third‐generation TKIs. In NSCLCs with single uncommon EGFR mutations, the mutations included G719X (*n* = 27), S768I (*n* = 7), and L861Q (*n* = 25). The remaining patients had NSCLCs with compound mutation types, of which G719X + L861Q was most frequently detected (*n* = 7). Five patients carried the G719X + E709X compound mutation and four patients (4.8%) carried the G719X + S768I compound mutation. Further details of the different mutation types in the NSCLC patients are given in Table 2.

**TABLE 1:** Baseline characteristics of all patients with uncommon EGFR mutations.

	Non‐small‐cell lung cancer patients (*n* = 84)	
Characteristics	No.	%
Sex		
Male	45	53.6
Female	39	46.4
Age		
Median	62	
Range	29–81	
≤65	53	63.1
>65	31	36.9
Stage		
III	4	4.8
IV	80	95.2
Smoking history		
Former	39	46.4
Never	45	53.6
Histology		
Squamous carcinoma	5	6.0
Adenocarcinoma	79	94.0
ECOG PS		
0	29	34.5
1	55	65.5
Previous surgery		
Yes	23	27.4
No	61	72.6
Previous radiotherapy		
Yes	36	42.9
No	48	57.1
Brain metastases		
Yes	34	40.5
No	50	59.5
Different generation TKIs		
Second‐generation TKIs	63	75.0
Third‐generation TKIs	21	25.0

Abbreviations: EGFR, epidermal growth factor receptor; ECOG PS, Eastern Cooperative Oncology Group Performance Status; TKIs, tyrosine kinase inhibitors.

**TABLE 2:** Different uncommon EGFR mutation subtypes in NSCLC patients.

	Mutation subtypes of patients (*n* = 84)	
Mutation genes	No.	%
Single mutation		
G719X	27	32.0
S768I	7	8.3
L861Q	25	29.8
Compound mutation		
G719X + E709X	5	6.0
G719X + L861Q	7	8.3
G719X + S768I	4	4.8
G719X + T790M	3	3.6
G719X + Her‐2	1	1.2
L861Q + L858R	1	1.2
L861Q + MET	1	1.2
S768I + L861Q	1	1.2
S768I + L858R	1	1.2
S768I + V769L	1	1.2

Abbreviations: EGFR, epidermal growth factor receptor; NSCLC, non‐small‐cell lung cancer.

### 3.2 Clinical efficacy

The ORR for all patients receiving TKIs was 47.6%, and the DCR was 86.9%. The ORR of patients treated with second‐generation TKIs was 54% and that of patients treated with third‐generation TKIs was 28.6%, which differed significantly (*P* = 0.044). In patients with the G719X mutation, the ORRs of the second‐ and third‐generation TKIs did not differ significantly (52.9% vs. 30%, respectively, *P* = 0.428). The ORRs of patients carrying the S768I mutation also did not differ significantly after treatment with second‐ or third‐generation TKIs (60% vs. 50%, respectively, *P* = 1.000). In patients with the L861Q mutation, the ORR for second‐generation TKIs was 55% (*n* = 11), which did not differ significantly from the ORR for third‐generation TKIs (20%; *P* = 0.327). In patients with compound mutations, ORR did not differ significantly after treatment with the second‐ or third‐generation TKIs (52.4% vs. 25%, respectively, *P* = 0.590). The detailed responses are listed in the Table 3. The DCRs of patients with mutations G719X, S768I, L861Q, or compound mutations who received second‐generation TKIs were 88.2%, 80%, 80%, and 90.5%, respectively, and those for patients receiving third‐generation TKIs were 90%, 50%, 100%, and 100%, respectively. Of the patients who received second‐generation TKIs, 34 achieved PR, whereas 6 of those who received third‐generation TKIs achieved PR. In both groups, only two patients showed PD, and these carried the mutation G719X or S768I. Among the patients receiving second‐generation TKIs, the responses of eight patients were still unclear. However, in only one patient who received a third‐generation TKI was the response to treatment unclear.

**TABLE 3:** Response rates of intent‐to‐treat NSCLC patients with uncommon EGFR mutations.

		G719X		S768I		L861Q		Compound mutation	
Different generation TKIs	Response rates	No.	%	No.	%	No.	%	No.	%
Second‐generation TKIs (*n* = 63)	Overall response	9	52.9	3	60	11	55	11	52.4
	Complete response	0	0	0	0	0	0	0	0
	Partial response	9	52.9	3	60	11	55	11	52.4
	Stable disease	6	35.3	1	20	5	25	8	38.1
	Progressive disease	1	5.9	0	0	0	0	0	0
	Unknown	1	5.9	1	20	4	20	2	9.5
Third‐generation TKIs (*n* = 21)	Overall response	3	30	1	50	1	20	1	25
	Complete response	0	0	0	0	0	0	0	0
	Partial response	3	30	1	50	1	20	1	25
	Stable disease	6	60	0	0	4	80	3	75
	Progressive disease	0	0	1	50	0	0	0	0
	Unknown	1	10	0	0	0	0	0	0

Abbreviations: NSCLC, non‐small‐cell lung cancer; TKIs, tyrosine kinase inhibitors.

### 3.3 Survival

**FIGURE 1:**
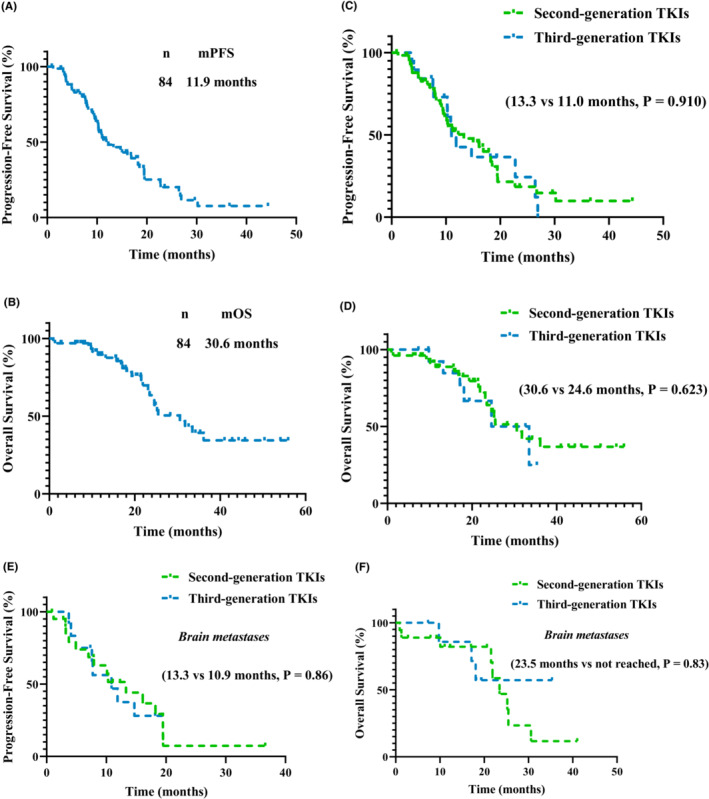
Kaplan–Meier estimates of progression‐free survival (PFS) and overall survival (OS). (A) PFS of the whole cohort of NSCLC patients with uncommon mutation including G719X, S768I, and L861Q (*n* = 84, PFS = 11.9 months); (B) OS of the whole cohort of NSCLC patients with uncommon mutation including G719X, S768I, and L861Q (*n* = 84, OS = 30.6 months); (C) PFS of patients with the second‐ and third‐generation TKIs not exhibited the difference (13.3 vs. 11.0 months, *P* = 0.910); (D) OS of patients with the second‐ and third‐generation TKIs not manifested the difference (30.6 vs. 24.6 months, *P* = 0.623); (E) No difference in PFS between the second‐ and third‐generation TKIs for NSCLC patients with brain metastases (13.3 vs. 10.9 months, *P* = 0.86); (F) No difference in OS between the second‐ and third‐generation TKIs for NSCLC patients with brain metastases (23.5 months vs. not reached, *P* = 0.83).

**FIGURE 2:**
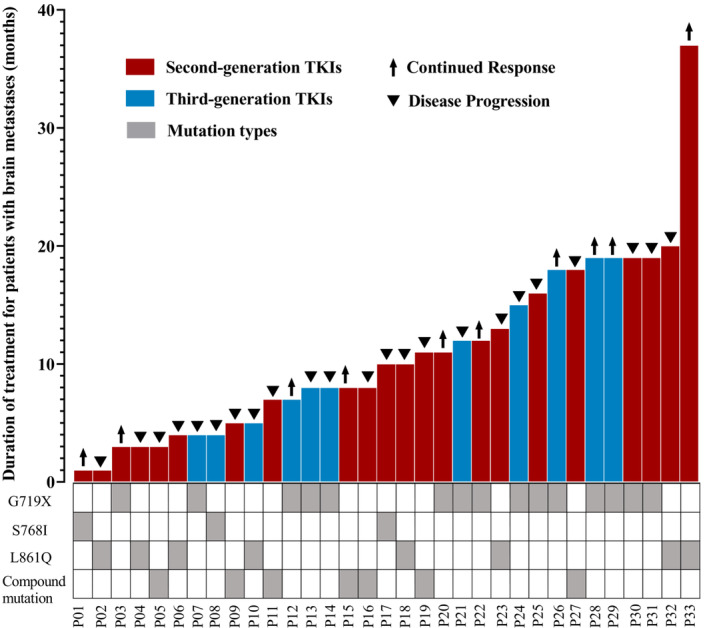
The swimmer's plot showing the PFS for all uncommon mutation, including G719X, S768I and L861Q, NSCLC patients with brain metastases.

**FIGURE 3:**
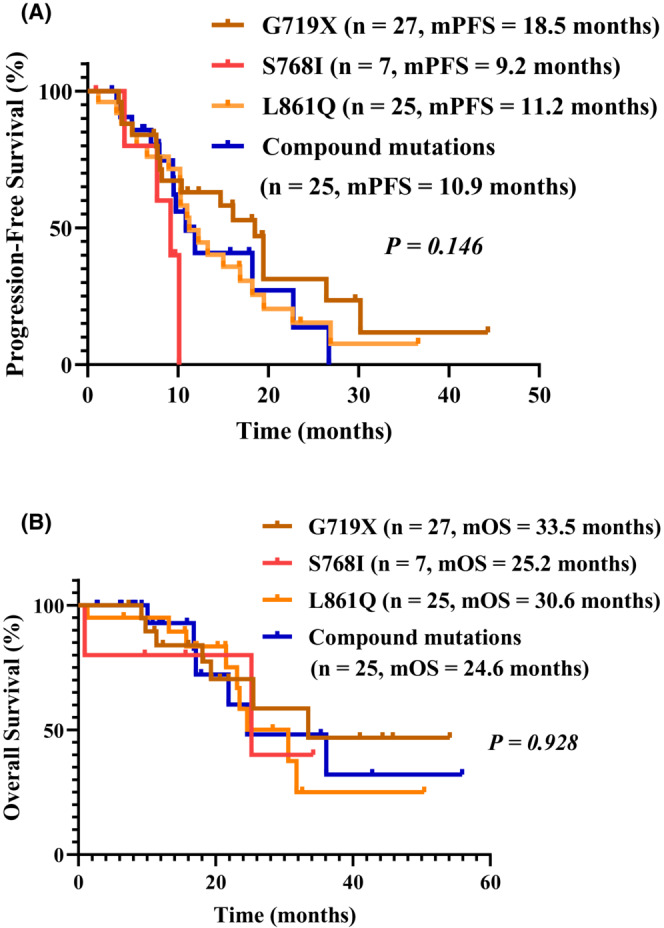
Kaplan–Meier estimates of progression‐free survival (PFS) and overall survival (OS). (A) No difference in PFS of different uncommon mutation types (G719X vs. S768I vs. L861Q vs. Compound mutations, 18.5 vs. 9.2 vs. 11.2 vs. 10.9 months, P = 0.146); (B) No difference in OS of different uncommon mutation types (G719X vs. S768I vs. L861Q vs. Compound mutations, 33.5 vs. 25.2 vs. 30.6 vs. 24.6 months, *P* = 0.928) .

The median PFS (mPFS) for all NSCLC patients with uncommon EGFRs mutation treated with TKIs was 11.9 months and the OS was 30.6 months (Figure 1A,B). The mPFS of the patients receiving second‐ and third‐generation TKIs did not differ significantly (13.3 vs. 11.0 months, respectively, *P* = 0.910) (Figure 1C); nor did OS (30.6 vs. 24.6 months, respectively, *P* = 0.623) (Figure 1D). Among patients with brain metastases, PFS did not differ significantly between those treated with second‐ or third‐generation TKIs (13.3 vs. 10.9 months, respectively, *P* = 0.86) (Figure 1E); nor did OS (23.5 months vs. not reached, respectively, *P* = 0.83) (Figure 1F). The responses of patients with brain metastasis receiving the different generation TKIs and their mutation types are shown in Figure 2. The responses of patients receiving third‐generation TKIs and their mutation types are shown in Figure S1. A multivariate analysis showed that treatment with a specific generation TKI was not a significant factor affecting PFS (*P* = 0.566, hazard ratio, 1.209; 95% confidence interval, 0.632–2.315) (Figure S2). PFS did not differ significantly between patients with different types of uncommon EGFR mutations (G719X, 18.5 months; S768I, 9.2 months; L861Q, 11.2 months; compound mutations, 10.9 months; *P* = 0.146) (Figure 3A). Patients with the G719X mutation showed longer OS than those with other types of mutation. However, OS did not differ significantly between the various mutation types (G719X, 33.5 months; S768I, 25.2 months; L861Q, 30.6 months; compound mutations, 24.6 months; *P* = 0.928) (Figure 3B).

### 3.4 Toxicity

The major AEs of patients receiving TKI therapies were diarrhea, rash, ulcer, paronychia, pruritus, abnormal liver function, and hand–foot syndrome. Of the patients treated with second‐generation TKIs, 10 (15.9%) experienced Grade 1 rash, 7 patients (11.1%) Grade 1 pruritus, and 4 patients (6.3%) Grade 1 diarrhea. Eight patients suffered Grade 1 ulcer, especially dental ulcer. Of the severe toxicities, 7 patients (11.1%) experienced Grade 3 rash and 1 patient (1.6%) Grade 4 rash. The occurrence rates for Grade 3 diarrhea and Grade 3 paronychia were 7.9% (*n* = 5) and 9.5% (*n* = 6), respectively. One patient presented with Grade 3 abnormal liver function. Among patients receiving third‐generation TKIs, no severe AEs (Grade 3 or 4) were reported. Five patients displayed Grade 1 rash (23.8%) and only 2 patients Grade 2 rash. One patient suffered Grade 2 diarrhea, and another Grade 2 pruritus. Only 1 patient (4.8%) displayed Grade 1 paronychia. No abnormal liver function or hand–foot syndrome was observed in patients receiving third‐generation TKIs. The details of all toxicities are shown in Table S1.

## 4 DISCUSSION

To the best of our knowledge, this study is the first to compare the efficacy and safety of second‐ and third‐generation TKIs for NSCLC carrying uncommon EGFR mutations, including G719X, S768I, or L861Q. We detected no difference in the efficacy of second‐ and third‐generation TKIs, suggesting that both therapy regimens are suitable for these patients if their toxicities are well tolerated.

Among the uncommon mutations of EGFR associated with NSCLC, G719X, S768I, and L861Q are sensitive mutations. The efficacy of therapies for patients with these types of NSCLC has been reported, but the sample sizes have been small. The report of Yang et al. showed that afatinib is more efficacious than first‐generation TKIs, such as erlotinib and gefitinib, for NSCLC with the G719X, S768I, or L861G mutation. A preclinical study of NSCLC models carrying uncommon EGFR mutations concluded that osimertinib is active against NSCLC with these mutations. Even the lowest concentration of osimertinib inhibited the relevant phosphorylation and blocked the growth of the tumors.^14^ A Phase II trial also preliminarily demonstrated the efficacy of osimertinib in NSCLC patients with uncommon EGFR mutations.^8^ In a previous case report, a 65‐year‐old woman with brain metastasis from a lung adenocarcinoma with the EGFR G719X and S768I mutations was treated successfully with osimertinib as the first‐line therapy, demonstrating the efficacy of this drug and its control of central nervous system symptom.^15^ Therefore, specific therapy regimens must be evaluated based on the different conditions of patients. In the present study, the PFS of patients with brain metastasis treated with second‐ or third‐generation TKIs did not differ significantly (13.3 vs. 10.9 months, respectively, *P* = 0.86); nor did their OS (23.5 months vs. not reached, respectively, *P* = 0.83). However, no patient receiving a third‐generation TKI had died at the end of the study period. Whether the third‐generation TKIs are more efficacious that second‐generation TKIs for NSCLC with brain metastasis must be investigated in future studies because this study was limited by its small sample size and its retrospective nature.

G719X is a mutation in exon 18 of EGFR, and G719S/A/C account for most G719X mutations.^16^ A previous study provided evidence that NSCLC patients with the G719X mutation of EGFR who received afatinib survived substantially longer than those with the L861G mutation, and that patients with mutation S768I had the longest survival of those with one of these three mutations.^6^ The median OS of all patients carrying the G719X mutation (*n* = 18) was 26.9 months (16.4‐NE) and that of patients carrying the L861G mutation (*n* = 16) was 17.1 months (15.3–21.6 months).^6^ The ORRs of patients with G719X, S768I, or L861Q receiving afatinib were 77.8% (*n* = 18), 100% (*n* = 8), or 56.3% (*n* = 16), respectively. Compared with the study of KCSG‐LU15‐09,^17^ the ORRs for patients with EGFR mutations G719X, S768I, or L861Q who received osimertinib were 53% (*n* = 19), 38% (*n* = 8), or 78%, respectively (*n* = 9). In our study, the ORRs for patients with EGFR mutations G719X, S768I, or L861Q who received second‐generation TKIs were 52.9% (*n* = 9), 60% (*n* = 3), or 55% (*n* = 11), respectively. Similarly, the ORRs of patients with EGFR mutation G719X, S768I, or L861Q treated with third‐generation TKIs were 30%, 50%, or 20%, respectively. Therefore, the ORRs of patients with the various uncommon mutation types to the two types of TKIs also did not differ significantly. The number of patients in the present study still less than the condition we expected.

Another case report suggested that compound mutations could promote to accomplish the biological tumorigenesis, so our results may indicate that compound uncommon EGFR mutations respond better to these TKIs than single uncommon EGFR mutations.^18^ Another brief report also demonstrated that the co‐occurrence of sensitive uncommon EGFR mutations with S768I was especially responsive to erlotinib, which was not observed in tumors with the single S768I mutation.^19^ However, the discrepancies in research results indicate that compound EGFR mutations correlate with poor prognoses.^20^ In the present study, neither PFS nor OS differed significantly between patients with compound or single mutations. The effects of the different uncommon EGFR mutation types in patients with NSCLC warrants further investigation.

In terms of the safety profiles observed in the present study, the third‐generation TKIs tended to show a lower incidence of severe toxicity than the second‐generation TKIs, consistent with previous studies. Eight of the patients receiving second‐generation TKIs required dose modification due to AEs. However, no treatment with the third‐generation TKIs was discontinued due to toxicity. According to the related researches, we find that the occurrence of adverse events may be associated with the germline polymorphisms toward the target of EGFR.^21^, ^22^, ^23^ And the polymorphisms may become the novel biomarkers to predict the occurrence of adverse events of patients received the therapy of TKIs.^22^ The more details could be explored in the future.

The limitations of our study should be taken into consideration. First, different institutions may detect different gene mutations. Second, both the first‐ and later‐line therapies were included in analysis. Different therapy lines should be divided into subgroups to determine whether the timing of treatment influences the outcome. Third, the number of NSCLC patients with uncommon EGFR mutations was small and should be higher in future studies. We appreciate that a prospective study of a larger sample is required to verify the outcomes reported here.

## 5 CONCLUSIONS

The efficacy and safety of second‐ and third‐generation TKIs for NSCLC with uncommon EGFR mutations do not differ significantly. Therefore, both regimens can be used to treat patients with EGFR with uncommon mutations, especially G719X, S768I, and L861Q.

## AUTHOR CONTRIBUTIONS

**Yue Hao:** Data curation (lead); investigation (lead); methodology (lead); software (lead); writing – original draft (lead). **Manyi Xu:** Investigation (equal); methodology (equal); software (equal); writing – original draft (equal). **Jianan Jin:** Data curation (equal); investigation (equal); software (equal). **Jinfei Si:** Data curation (equal); investigation (equal); validation (equal); visualization (equal). **Chunwei Xu:** Conceptualization (equal); funding acquisition (equal); project administration (equal); supervision (equal); writing – review and editing (equal). **Zhengbo Song:** Conceptualization (equal); funding acquisition (equal); project administration (equal); resources (equal); supervision (equal); writing – review and editing (equal).

## FUNDING INFORMATION

The study was funded by the Medical Scientific Research Foundation of Zhejiang Province (No. 2022KY653). This study was sponsored by a grant from the Zhejiang provincial program for the Cultivation of High‐level Innovative Health talents (to Zhengbo Song). The study was also supported in part by grants from the Medical Scientific Research Foundation of Zhejiang Province of China (2023KY666).

## CONFLICT OF INTEREST STATEMENT

All authors declare no conflict of interest.

## ETHICS APPROVAL AND CONSENT TO PARTICIPATE

Approval of the study protocol was obtained from Zhejiang Cancer Hospital Institutional Review Board Committee (approval number: IRB‐2021‐440). All patients Individual consent for this retrospective analysis was waived.

## Supporting information


Figure S1.

Figure S2.

Table S1.

## Data Availability

The datasets used and/or analyzed during the current study are available from the corresponding author upon reasonable request.
