# Supplementary figures and images for: Comparison of efficacy and safety of second‐ and third‐generation TKIs for non‐small‐cell lung cancer with uncommon EGFR mutations
Source: Cancer Med. 2023 Jun 12;12(15):15903–11. doi: 10.1002/cam4.6229 (PMC10469645; doi:10.1002/cam4.6229)

Supplementary Figure 1 (S1)

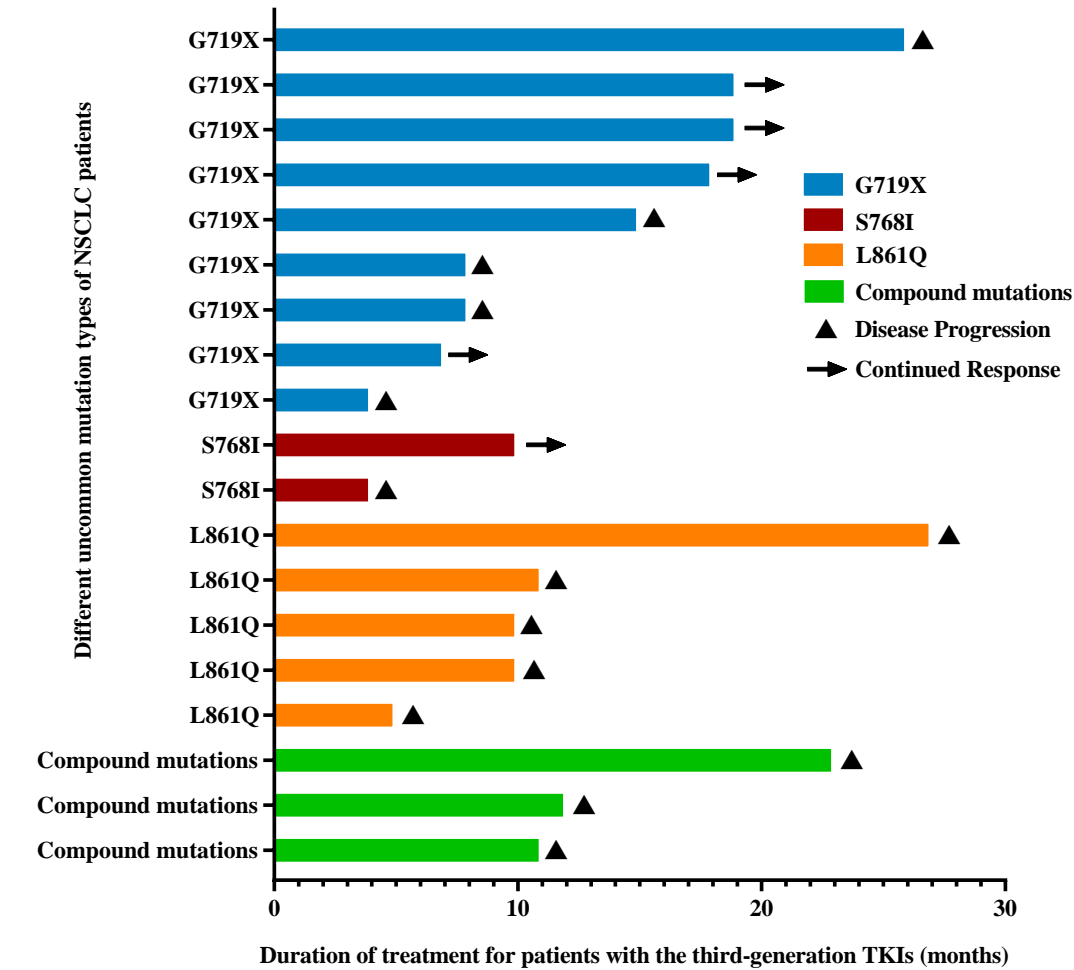

Supplement: Supplementary file 1 — Figure S1. [file CAM4-12-15903-s002.pdf]

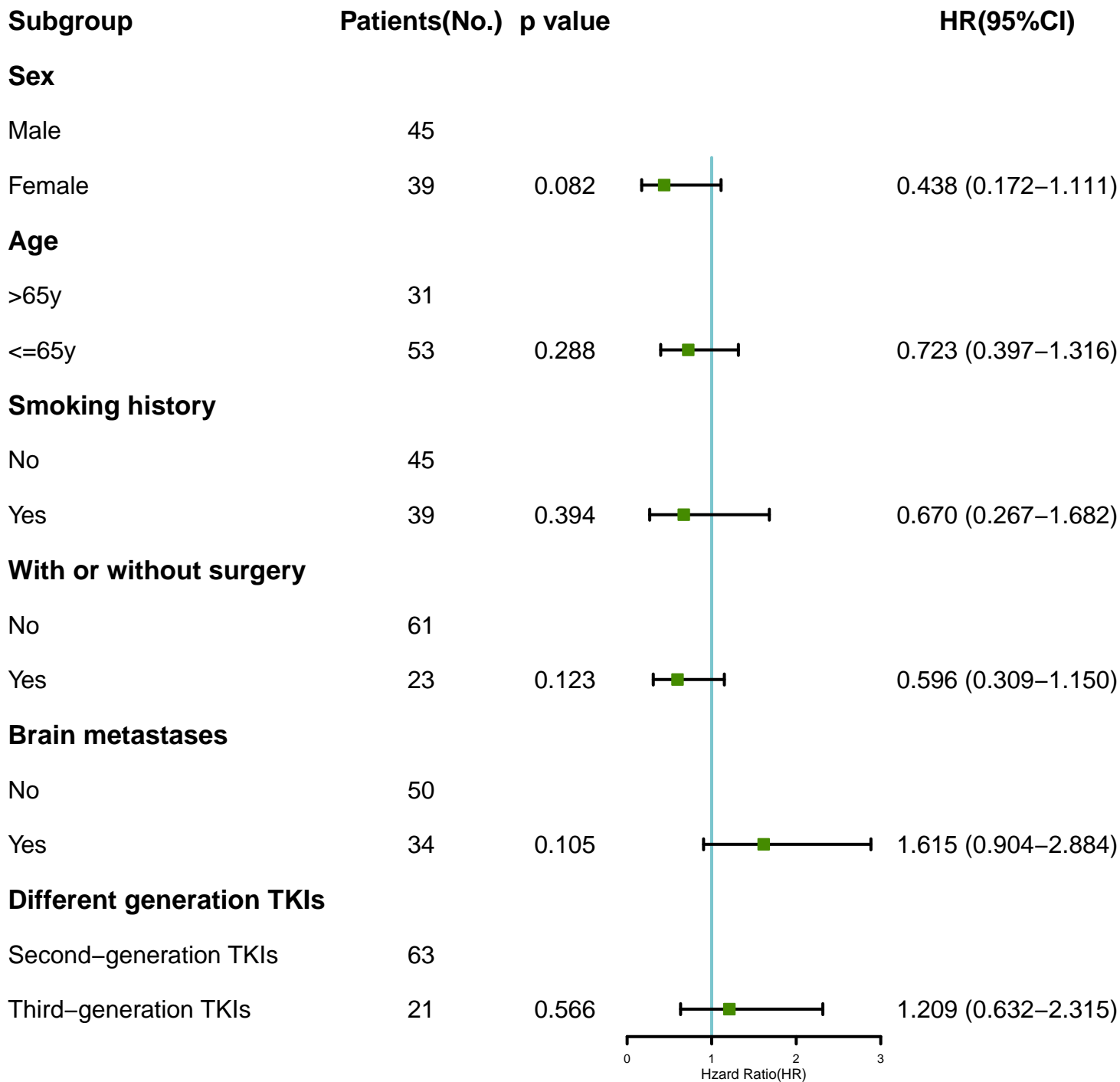

Supplement: Supplementary file 2 — Figure S2. [file CAM4-12-15903-s001.pdf]
