# Supplementary material for: Comparison of efficacy and safety of second‐ and third‐generation TKIs for non‐small‐cell lung cancer with uncommon EGFR mutations
Source: Cancer Med. 2023 Jun 12;12(15):15903–11. doi: 10.1002/cam4.6229 (PMC10469645; doi:10.1002/cam4.6229)
Supplement: Supplementary file 3 — Table S1. [file CAM4-12-15903-s003.docx]

**Supplementary material**

**Supplementary Table 1**

Adverse events of TKIs in NSCLC patients with uncommon EGFR mutations

| **Different generation TKIs** | **Adverse events** | **Grade 1** | **Grade 2** | **Grade 3** | **Grade 4** |
| --- | --- | --- | --- | --- | --- |
| Second-generation TKIs | Diarrhea | 4 (6.3%) | 2 (3.2%) | 5 (7.9%) | 0 |
| (n = 63) | Rash | 10 (15.9%) | 3 (4.8%) | 7 (11.1%) | 1 (1.6%) |
|  | Ulcer | 8 (12.7%) | 1 (1.6%) | 1 (1.6%) | 0 |
|  | Paronychia | 3 (4.8%) | 1 (1.6%) | 6 (9.5%) | 0 |
|  | ALF | 1 (1.6%) | 0 | 1 (1.6%) | 0 |
|  | Pruritus | 7 (11.1%) | 1 (1.6%) | 0 | 0 |
|  | HFS | 3 (4.8%) | 2 (3.2%) | 1 (1.6%) | 0 |
| Third-generation TKIs | Diarrhea | 2 (9.5%) | 1 (4.8%) | 0 | 0 |
| (n = 21) | Rash | 5 (23.8%) | 2 (9.5%) | 0 | 0 |
|  | Ulcer | 1 (4.8%) | 0 | 0 | 0 |
|  | Paronychia | 1 (4.8%) | 0 | 0 | 0 |
|  | ALF | 0 | 0 | 0 | 0 |
|  | Pruritus | 4 (19%) | 1 (4.8%) | 0 | 0 |
|  | HFS | 0 | 0 | 0 | 0 |

Abbreviations: NSCLC, non-small cell lung cancer; TKIs, tyrosine kinase inhibitors; ALF, abnormal liver function; HFS, hand-foot syndrome.
